# A Bond-Wire Drift Offset Minimized Capacitance-to-Digital Interface for MEMS Accelerometer with Gain-Enhanced VCO-Based Quantization and Nested Digital Chopping Feedback Loops

**DOI:** 10.3390/s21144627

**Published:** 2021-07-06

**Authors:** Fanyang Li, Tao Yin, Haigang Yang

**Affiliations:** 1 Fujian Province Integrated Circuit Design Center, Fuzhou University, Fuzhou 350108, China; t12046@fzu.edu.cn; 2 Institute of Semiconductors, Chinese Academy of Sciences, Beijing 100083, China; yint@semi.ac.cn; 3 School of Microelectronics, University of Chinese Academy of Sciences, Beijing 100049, China; 4 Shandong Industrial Institute of Integrated Circuits Technology Ltd., Jinan 250001, China; 5 Aerospace Information Research Institute, Chinese Academy of Sciences, Beijing 100094, China

**Keywords:** MEMS, accelerometer, VCO, digital chopping, offset, bond-wire

## Abstract

This paper presents an output offset minimized capacitance-to-digital interface for a MEMS accelerometer. With a gain-enhanced voltage-controlled oscillator (VCO)-based quantization loop, the interface is able to output a digital signal with improved dynamic range. For optimizing the output offset caused by nonideal factors (e.g., the bond-wire drift), a nested digital chopping feedback loop is embedded in the VCO-based quantization loop. It enables the interface to minimize the output offset without digital filtering and digital-to-analog conversion. The proposed architecture is well suited for dynamic range and offset improvements with low cost. Fabricated with a 0.18 μm Global Foundry (GF) CMOS process, the interface offers a 78 dB dynamic range with 0.4% nonlinearity from a single 2 V supply. With the input referred offset up to 1.3 pF, the offset cancellation loop keeps the DC output offset within 40 mV. The power dissipation is 6.5 mW with a bandwidth of 4 kHz.

## 1. Introduction

Capacitive micro-accelerators [1,2] have gained popularity in numerous applications ranging from the microgravity measurement to the self-contained navigation and guidance because of low power and high sensitivity characteristics.

For signal readout, voltage-controlled force approaches are reported in [3,4,5,6]. Nevertheless, the approach has the drawback of low linearity. The drawback results from the nonlinearity existing in voltage-to-force transduction due to the MEMS actuation capacitor mismatch. A common way to overcome it is to translate programmable DC voltage into the force voltage to balance the seismic mass according to the mismatch. However, complicated digital algorithms or auxiliary analog circuits are needed to achieve balance, so the design is complicated. In order to directly output a digital signal and improve linearity, a high-precision capacitive accelerometer typically operates in a closed loop [7,8,9], which includes a ΔΣ analog-to-digital convertor (ADC). However, the extra ΔΣ ADC consumes considerable power and makes the design complicated.

To overcome the above-mentioned nonlinearity induced by the mismatch with a simpler topology, a pulse width modulation force (PWM) or ΔΣ feedback loop approach in [10,11,12,13,14,15,16] is presented. These are able to eliminate the nonlinearity in voltage-to-force transduction. In [15,16], a pulse-width-modulation force feedback approach was proposed. However, extra power was induced by designing an extra oscillator, and the PWM signal had to be converted into a digital signal again for digital signal processing (DSP). To directly output a digital signal, a ΔΣ feedback loop approach [10,11,12,13,14,17] was proposed. In this approach, conventional integrators designed with operational trans-conductance amplifiers (OTAs) are commonly used. Although higher order noise shaping can be achieved by adding extra OTAs, the increased number of OTAs seriously degrades power performance. Additionally, the output dynamic range is limited because of OTA transistor saturation. For a wider dynamic range, a higher feedback supply voltage is needed [5,17].

In addition, an output offset caused by nonideal factors [16,18] (e.g., the bond-wire capacitance drift) is considerable. To address the issue, an offset minimization loop with electrostatic spring constant modulation was proposed in [14]. Although the approach was able to minimize the offset by the loop, it needed decimators and band-pass digital filters to tease out the feedback DC and AC modulation signal and its harmonic components. To facilitate the system design, an off-chip FPGA was needed. Moreover, since the FPGA outputs digital signals, a multi-bits DAC (digital-to-analog converter) with a trans-impedance amplifier (TIA) had to be designed.

In this paper, we propose a novel readout interface with a simple gain-enhanced VCO-based quantization loop. The interface outputs a dynamic range with an improved digital signal and optimized power performance. Simultaneously, with a simple nested digital chopping feedback loop, it minimizes the DC output offset without the digital filters and a multibits DAC, so the dynamic range can be further enlarged. Section 2 describes the proposed interface. Section 3 focuses on the circuit implementation. Results are given in Section 4. The conclusion is given in Section 5.

## 2. The Proposed Interface Principle

### 2.1. Challenges Based on MEMS Characteristics

**Figure 1:**
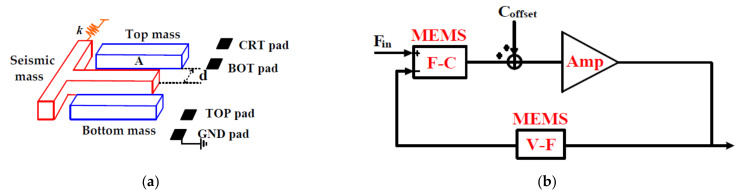
(**a**) MEMS architecture. (**b**) Common model of MEMS and interface signal processing.

The MEMS architecture is shown in Figure 1a. The seismic mass controlled by the spring is placed between the top and bottom masses. The top, bottom and seismic mass voltage pads are named TOP, BOT and CRT, respectively, and can be equalized as two sensing capacitors. The capacitor variation can be implemented by the input and feedback forces. The input force-to-capacitor transfer function depends on the spring coefficient *k*, which is determined by the common mode voltage of the two capacitors. With regard to the feedback voltage-to-force conversion, the TOP and BOT pads are connected to the feedback voltage and the feedback to the opposite one. According to the architecture and its characteristics, the common model of MEMS and interface signal processing is shown in Figure 1b: The interface can be seen as an amplifier with a gain of G_1_. The MEMS can be divided into F-C and V-F blocks [14]. The transfer functions of the two blocks can be approximately expressed as follows:

$$H_{F-C}\approx \alpha_{0}/k$$

$$H_{V-F}=\varepsilon AV_{\sup ply}/d^{2}$$

where α_0_ is the displacement-to-capacitance gain, *k* is the spring coefficient, A is the mass area and d is the nominal gap between the plates. According to the equations above, the transfer coefficients of F-C and V-F blocks can be tuned by the spring coefficient *k* and the force feedback supply voltage. The *k* value depends on the top and bottom masses common voltage [14].

With the model, the output voltage induced by the input force can be given as follows:

$$V_{out}=F_{in}(\alpha_{0}/k)G_{1}/[1+(\alpha_{0}/k)(\varepsilon AV_{\sup ply}/d^{2})G_{1}]$$

The dynamic range depends on the amplifier gain. With the output signal amplitude increasing, the gain degrades because of the output stage transistors entering the linear region. Therefore, the dynamic range performance deteriorates. To improve the dynamic range, the feedback coefficient value can be increased by means of a higher force feedback supply voltage [17]. Thus, the input force dynamic range can be improved.

On the other hand, with a bond-wire offset, the output offset voltage induced by the bond-wire offset can be expressed as follows:

$$V_{out}=C_{offset}G_{1}/[1+(\alpha_{0}/k)(\varepsilon AV_{\sup ply}/d^{2})G_{1}]$$

The above equation shows that the bond-wire offset is able to induce the offset output voltage. Moreover, by varying *k*, the offset voltage can be correspondingly changed with a certain gain so that the dynamic range is limited. Combined with the equations above, it is known that if the top and bottom masses common voltage is modulated by a periodic digital signal, a periodic output offset is also produced [14].

### 2.2. The Proposed Interface

**Figure 2:**
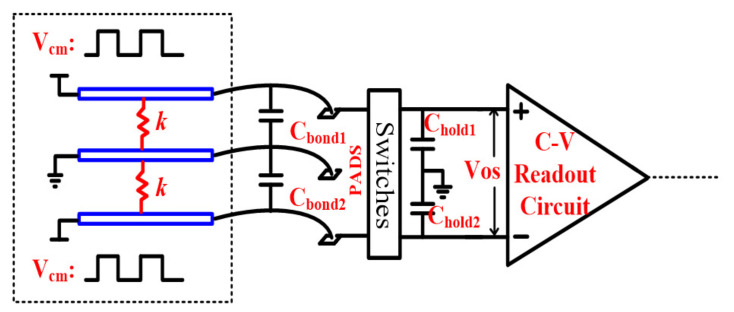
The MEMS F-C and interface connection.

With the MEMS undertaking the spring parameter *k* modulation and connection with the interface shown in Figure 2, the interface input referred offset and parameter *k* can be expressed as follows:

$$C_{referred-offset}=\Delta C_{bond}+\Delta C_{hold}$$

$$k_{m}(t)=aV_{cm}^{2}(t)$$

where ΔC_bond_ and ΔC_hold_ are induced by the bond-wire drift parasite and hold capacitors mismatch, respectively. Vcm(t) is the common mode voltage of two actuation capacitors in the MEMS. a is the modulation coefficient. With the modulated feedback coefficient [14], the output offset can be converted into DC and AC. If the ratio of AC and DC can be designed to be at an ultra-low level, the output AC offset can be ignored. Thus, the dynamic range is maintained if the DC offset is minimized.

**Figure 3:**
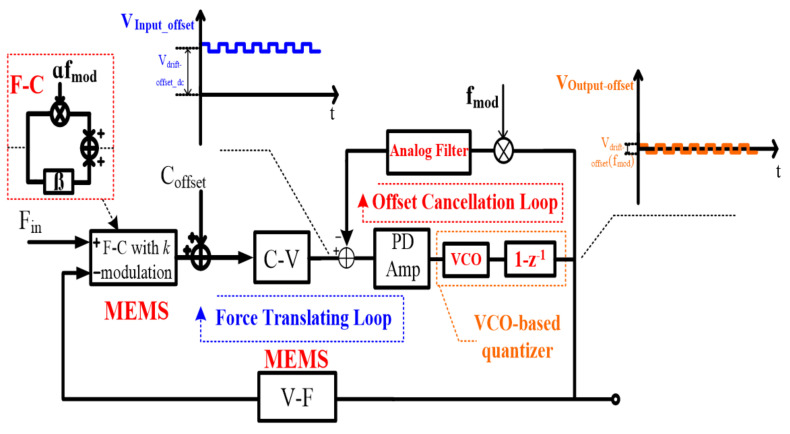
Illustration of the proposed interface.

With the ideology above, the corresponding proposed interface topology is shown in Figure 3. It can be divided into two parts: a gain-enhanced VCO-based quantization loop and a nested digital chopping feedback loop. The gain-enhanced VCO-based quantization loop processes the force signal. It is constituted by a F-C function block (MEMS), a C-V readout circuit, a PD amplifier, a gain-enhanced VCO-based quantizer and a V-F function block (MEMS). The nested digital chopping feedback loop is nested in the force translation loop to minimize the output offset. It consists of the PD amplifier, the VCO-based quantizer, a digital chopper and an analog filter.

With regard to the force signal, since this is not modulated by *k* modulation while being modulated by the digital chopping signal (the same frequency with *k* modulation) in the nested digital chopping feedback loop, the signal can be transferred to the high frequency range and filtered out by the analog filter. Therefore, to process the force signal throughout the gain-enhanced VCO-based quantization loop, the nested digital chopping feedback loop is not appropriate. On the other hand, as shown in Figure 3, offset drift minimization can be achieved because in the nested digital chopping feedback loop, the output AC offset is again modulated by the digital chopping signal and converted to a DC offset with a certain gain by the analog filter. Then the DC offset is fed back to minimize the output DC offset shown in Figure 3.

### 2.3. The Gain-Enhanced VCO-Based Quantization Loop

**Figure 4:**
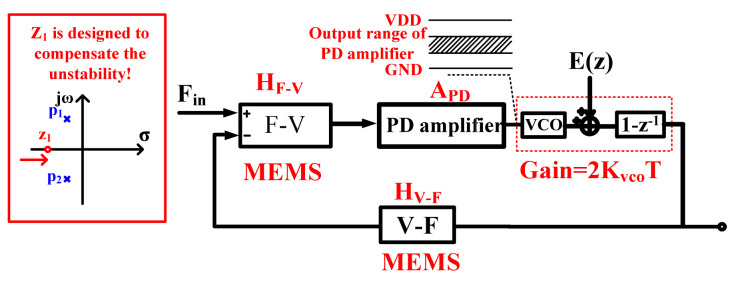
Principle of the force translating loop.

The gain-enhanced VCO-based quantization loop is shown in Figure 4: Considering two poles existing in the MEMS, a PD amplifier is designed in order to compensate loop stability, and the quantization noise is further shaped by the VCO-based quantizer. According to the force translating loop, the output signal can be approximately expressed as follows:

$$V_{out\_force}\left(f_{in}\right)=\frac{H_{F-V}A_{PD}2K_{vco}T}{1+H_{V-F}A_{F-V}A_{PD}2K_{vco}T}F(f_{in})+\frac{2f_{in}T}{H_{V-F}A_{F-V}A_{PD}2K_{vco}T}E(f_{in})$$

where *H_F-V_* and *A_PD_* are the gain of the F-V converter (a model of MEMS F-V and readout circuit) and PD amplifier, *H_V-F_* is the feedback coefficient of the force transferring loop, *K_vco_* is the frequency gain coefficient and *T* is the sampling frequency. Unlike conventional interfaces, the quantization noise in the proposed interface is improved by *f_in_/K_vco_* by the gain-enhanced quantizer without designing more OTAs (used as the integrator), so fewer nondominant poles are induced and the bandwidth and power can be improved. With the zero designed at about 100 Hz, a bandwidth of 4 kHz with the phase margin π/4 is achieved.

On the other hand, unlike the dynamic range limited by the OTA in conventional approaches, with the gain of the VCO-based quantizer designed to be 20 dB, the output dynamic range can be extended near the rail-to-rail level without consideration of the OTA dynamic range, and the power sacrificed to improve the input force dynamic range can also be further saved.

### 2.4. The Nested Digital Chopping Feedback Loop

**Figure 5:**
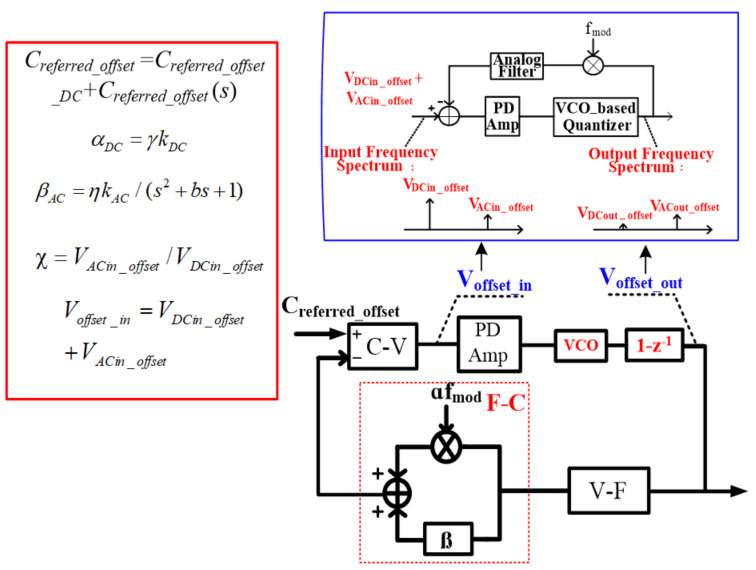
Principle of the offset minimization loop.

With regard to the two actuation capacitors modulated by the common voltage, the equivalent DC and AC MEMS F-V transfer functions shown in Figure 5 can be given by:

$$\alpha_{DC}=\gamma k_{DC}$$

$$\beta_{AC}=\eta k_{AC}/(s^{2}+bs+1)$$

where γ and η are the DC and AC coefficients of the modulated spring parameter *k*. According to the equations above and topology shown in Figure 5 above, the output offset can be expressed as follows:

$$V_{offset\_out}=\frac{K_{Q}}{1+\alpha F_{V-F}GK_{Q}}*\frac{1}{\chi A_{ana\log}}C_{referred-offset\_DC}+\frac{K_{Q}}{1+\beta F_{V-F}GK_{Q}}C_{referred-offset}(s)$$

where C_referred-offset_DC_ and C_referred-offset_(s) are the input-referred DC and AC capacitor offsets, respectively, G is the gain of the readout circuit and the PD amplifier, K_Q_ is the gain of the PD amplifier and quantizer, χ is the input DC and AC voltage offset ratio and *A_analog_* is the DC gain of the integrator. According to the equation above, the DC output offset is minimized and the dynamic range of the interface is enlarged by the digital chopping modulation signal f_mod_ and the analog filter in the offset minimization loop, as illustrated in Figure 5. For DC output offset optimization, *A_analog_* should be designed to be as high as possible. In order to obtain the optimized dynamic range, the ratio of the input DC and AC voltage offset should be increased as much as possible, and because of the VCO-based quantizer, the quantization noise is shaped and transferred to the high frequency range by the force-translating loop. Since the frequency range of the shaped quantization noise is located in the range of 10^5^~10^6^ Hz, by means of the analog filter, the DC output offset is not interrupted by the noise.

On the other hand, because the modulation signal is a pulse waveform, the *k* modulation signal has considerable components at the odds harmonic frequencies. According to the above equation, because of the MEMS high-Q characteristic and PD amplifier, the components would be amplified with a certain gain. To suppress the components, the low-pass bandwidth of the PD amplifier itself is designed to be about 6 kHz (detailed in Section 4. D) and the *k* modulation frequency is set at 3 kHz.

## 3. Circuit Implementation

### 3.1. Circuit Implementation of k Modulation

**Figure 6:**
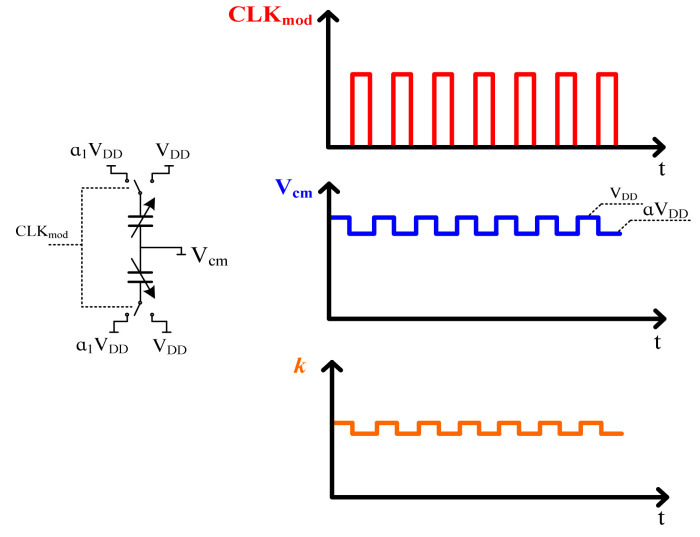
Spring k modulation implementation.

According to the discussion above, the spring parameter *k* modulation can be achieved by modulation of the supply voltages *V_DD_* and α_1_*V_DD_* [13], as shown in Figure 6. So, with supply voltage modulation, the AC and DC input offset ratio can be given by:

$$Ratio\approx [{\left(V_{\mathrm{DD}}\right)}^{2}-{\left(\;a_{1}V_{\mathrm{DD}}\right)}^{2}]/[{\left(V_{\mathrm{DD}}\right)}^{2}+{\left(\;a_{1}V_{\mathrm{DD}}\right)}^{2}]$$

Therefore, for the ratio α_1_ designed to be about 1/20, the accelerometer is correspondingly modulated by the supply voltages 1.9 V and 2 V, respectively.

### 3.2. Implementation of C-V Switched Capacitors

**Figure 7:**
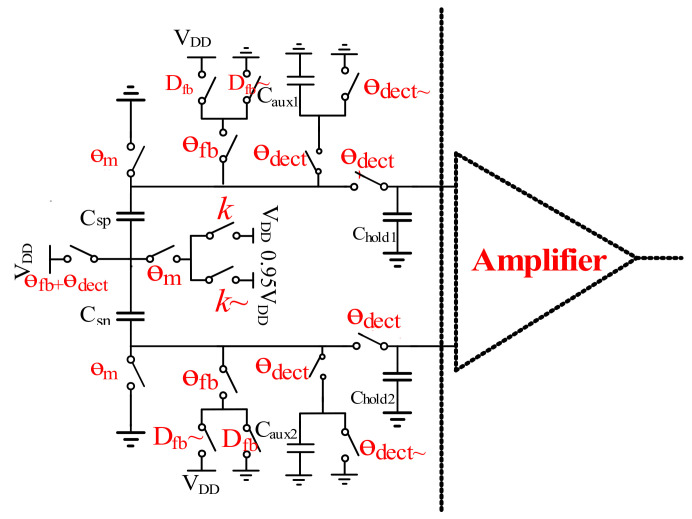
Topology of the C-V switched capacitors.

**Figure 8:**
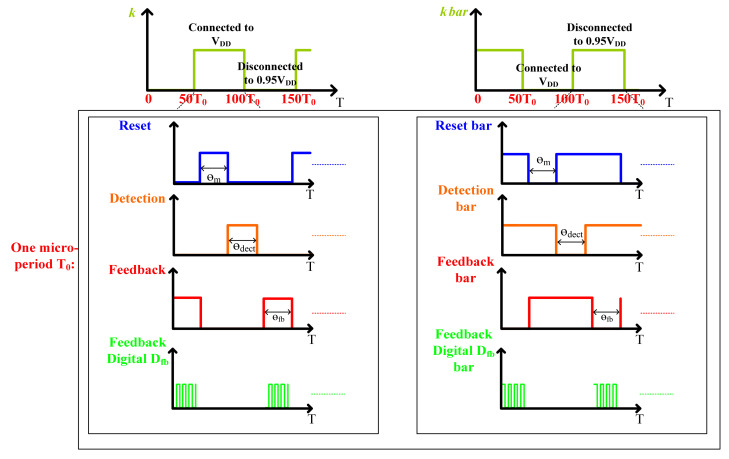
Timing diagram of the switched capacitors.

The C-V switched capacitors topology is shown in Figure 7. The accelerometer includes two parts: one includes the actuation and auxiliary capacitors C_sp,sn_ and C_aux1,2_, and the other the hold capacitors C_hold1,2_. With two auxiliary capacitors and switches, the capacitors C_sp,sn_ variation can be converted into the voltage signal. The hold capacitors C_hold1,2_ sample the voltage signal, which is amplified by the amplifier shown in Figure 7 for the following blocks. Figure 8 shows two corresponding timing diagrams: a macroperiod and a microperiod. In one single macroperiod, certain microperiods are included. In this design, the micoperiod T_0_ is designed to be 3.3 μs, while the macro-period is set at 100 T_0_. Thus, the macroperiod is 330 μs.

**Figure 9:**
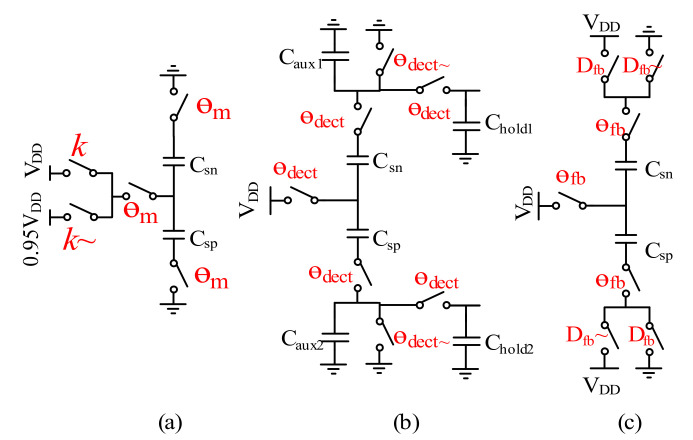
(**a**) Reset phase; (**b**) Signal detection phase; (**c**) Force feedback phase.

With regard to the macroperiod, the outputs of the actuation capacitors C_sp,sn_ are alternatively connected to V_DD_ (or 0.95V_DD_), as shown in Figure 8. The connection to V_DD_ (or 0.95V_DD_) is determined by the spring constant modulation signal. So, the spring constant can be expressed by:

$$k_{m}(t)=\delta *{\left[(\theta_{m}/2\pi )V_{cm}(t)\right]}^{2}$$

where δ is a coefficient constant and θ*_m_* is the reset phase in the microperiod. In the phase, the two capacitors are connected to the common mode voltage V_cm_(t), and V_cm_(t) is controlled by the modulation signal *k*. In the microperiod, three phases are used. The operation timing phases in one period are divided as the reset phase, signal detection and force feedback. In the reset phase, as in Figure 9a, the two actuation capacitors C_sp,sn_ are both connected to the common voltage, so the value of the spring parameter *k* can be modulated. In the signal detection phase, the auxiliary capacitors C_off_ are designed to measure the two actuation capacitors C_sp,sn_ variation. The time for the phase is θ_dect_. During θ_dect_, the actuation capacitors C_sp,sn_ variation can be obtained by the voltage share of the capacitors C_sn,sp_ and C_aux1,2_. The hold capacitors C_hold1,2_ sample the voltage variation, as shown in Figure 9b. In the force feedback phase, the charge of the two actuation capacitors is regulated by the digital feedback signal D_fb_, as shown in Figure 9c. According to the characteristic of the MEMS, the feedback force is given by:

$$F_{feedback}=\zeta (\theta_{fb}/2\pi )*V_{out}(s)\left|{}_{s=\omega_{in}}\right.$$

where ζ is a feedback coefficient constant, θ_fb_ is the force feedback phase and V_out_(s) is the output delta-sigma modulation signal.

### 3.3. The Readout Circuit

**Figure 10:**
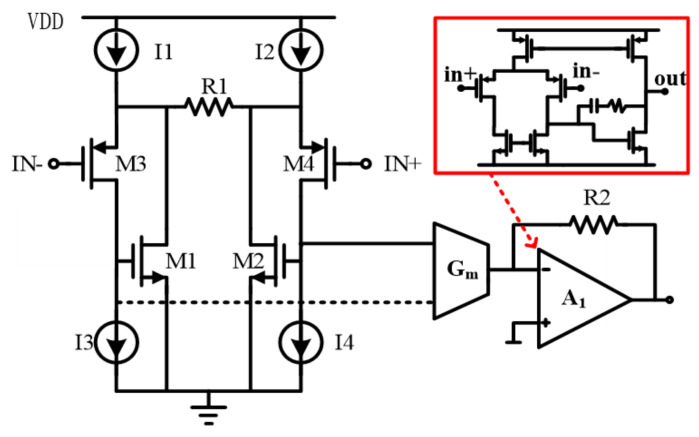
Topology of the readout circuit.

As shown in Figure 10, the readout circuit is constituted by a V-I input stage succeeded by an amp with a feedback resistor R_2_. The input stage mainly consists of M_1,3_ (or M_2,4_), R_1_ and a current mirroring G_m_. The M_1,3_ (or M_2,4_) and R_1_ play a role as a micro feedback loop. With the loop, the input voltage signal is converted into the current by R_1_. The current is transferred to the amp by mirroring of M_1,2_ currents with the G_m_. According to the topology above, the amplifier transfer function can be given by:

$$H(s)=(R_{2}/R_{1})/[1+(s/\omega_{0})]$$

where *ω*_0_ is the pole generated by the amp. The amp A_1_ is implemented with typical two-stage topology. The bandwidth of the whole amplifier is designed to be 60 kHz with a gain of 40 dB.

### 3.4. The PD Amplifier

**Figure 11:**
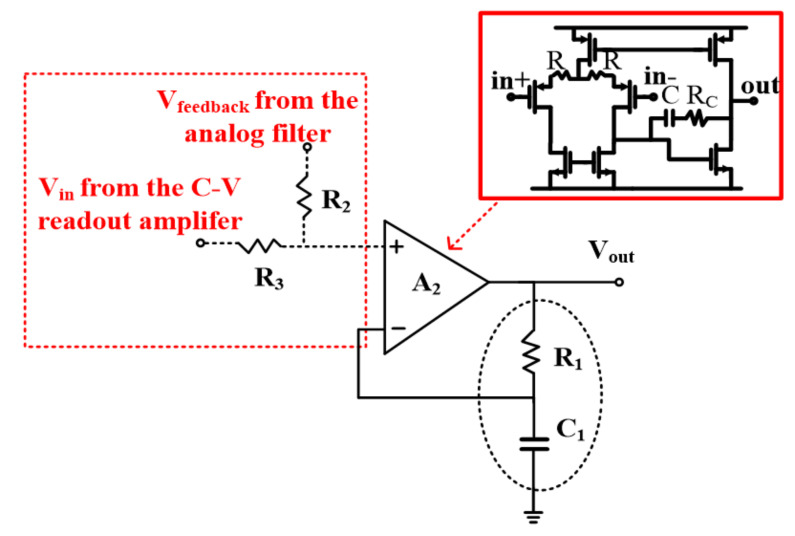
PD stabilization implementation.

With consideration of the AC characteristic of the accelerometer, since two conjugate poles exist in the accelerometer [2], an extra zero ω_z_ should be designed to compensate the phase margin of the loop. In order to generate the extra zero, a PD amplifier A_2_ with a frequency compensation network is designed, as shown in Figure 11. The amplifier A_2_ is also a typical two-stage amplifier with resistance degradation feedback. With the amplifier A_2_, the transfer function of the interface can be rewritten as follows:

$$H(s)=\frac{A_{open}(s)}{1+(1+R_{1}C_{1}s)A_{open}(s)}$$

where A_open_(s) is the amplifier open loop gain and R_1_ and C_1_ are the passive devices of the feedback network, which play the role in providing the zero *ω*_z_. A_open_(s) is a first order low pass filter. The corresponding pole ɷ_0_ suppress high frequency signals and has the following characteristic:

$$A_{open}(s)\left|{}_{s=\omega_{0}}\right.=1+R_{1}C_{1}s\left|{}_{s=\omega_{0}}\right.$$

According to the equation above, with the regulation of the resistor R_1_ and capacitor C_1_ shown in Figure 11, the pole *ω*_0_ is set to be 6 kHz to suppress the modulation harmonic components.

### 3.5. Gain-Enhanced VCO-Based Quantizer

**Figure 12:**
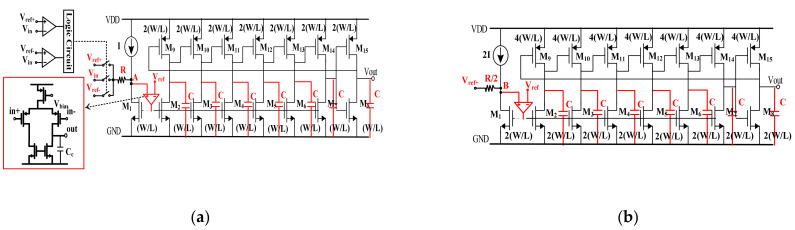
(**a**) Schematic of the proposed VCO; (**b**) schematic of the oscillator as the clock generator.

The gain-enhanced VCO-based quantizer can be divided into two parts: One part is the VCO and clock generator, and the other is a digital differentiator. Figure 12a shows a schematic of the proposed VCO. It consists of a VCO, comparators, V-I conversion resistor R and three voltage references selected by the switches. The VCO is a seven-stage ring oscillator with a center frequency of approximately 0.25 MHz. The voltage signal can be converted into the current signal by the resistor R. In order to achieve the low input impedance characteristic of the node A, the transistor M_1_ and micro-amp shown in Figure 12a constitute the feedback loop. The microamp is implemented with a typical single stage, and the capacitor C_C_ is designed to achieve the loop stability. With this topology, the frequency gain coefficient of the proposed VCO can be expressed as follows:

$$K_{VCO}=1/(7*V_{DD}*C*R)$$

where V_ref-_ < V_in_ < V_ref+_; V_DD_ is the supply voltage and C is the loading capacitor of the inverter cell. To keep the oscillation, another requirement has to be met and given as follows:

$$(V_{ref}-V_{ref-})/R=I$$

where the current I is the current source shown in Figure 12a.

Figure 12b shows the schematic of the sampling frequency oscillator. The oscillator also consists of the oscillator, V-I conversion resistor, and the voltage reference V_ref+_. The oscillator provides the sampling clock of the three D flip-flops in the digital differentiator. In order to achieve the antialias characteristic, the sizes of the transistors in the oscillator are designed to be twice that in the VCO, and the current source and resistor are designed to be 2I and R/2, respectively. According to the topology above, the gain of the quantizer can be expressed as follows:

$$G=V_{DD}/(V_{ref+}-V_{ref})$$

In this work, to achieve 20 dB gain, the voltage references V_ref+_, V_ref_ and V_ref-_ are designed to be 1.1 V, 1 V and 0.9 V, respectively, and the current source I and resistor R shown in Figure 12a are designed to be 5 μA and 20 kohm, respectively.

### 3.6. Analog Filtering in the Proposed Digital Chopping Feedback Loop

**Figure 13:**
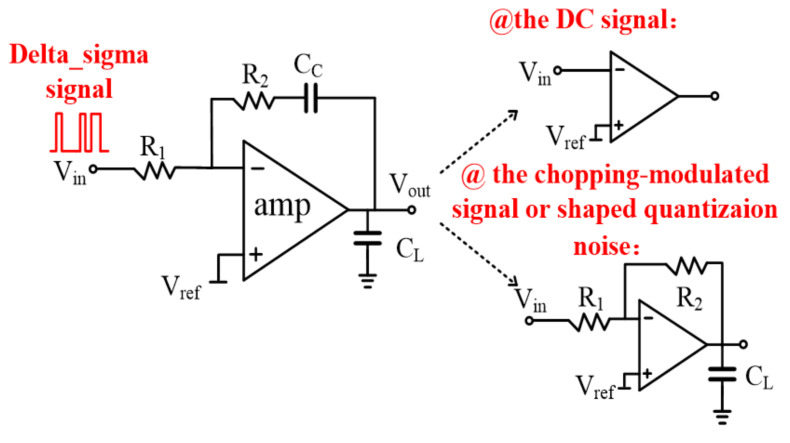
Analog filtering implementation.

Figure 13 is a complete topology of the analog filter designed in the offset cancellation loop. It is mainly constituted by the passive feedback network (C_C_ and R_1,2_) and the amplifier amp. With regard to the filtering characteristic, R_1_ and C_C_ provide a low pass cut-off frequency *f*_−3dB_. The loading capacitor C_L_ is designed to continuously filter out the high frequency signal. Therefore, as the input signals, the DC signal can be amplified while the chopping modulated signal, and the shaped quantization noise at the high frequency range, can be filtered out.

According to the analog filtering implementation, the loading capacitor C_L_ should be designed to make the filter an ideal integrator. With the design of R = 1/G_m_,C_C_ = C_L_ in this work_,_ the transfer function of the analog filtering can be given approximately by:

$$A(s)=\frac{A_{amp}}{1+A_{amp}R_{1}C_{C}s}$$

where G_m_ is the amplifier trans-conductance and A_amp_ is the amplifier DC open loop gain. The equation above shows that in order to filter out the chopping modulated signal, the capacitor C_C_ has to be large enough to make the dominant pole of the loop near DC.

## 4. Measurement Results and Analysis

**Figure 14:**
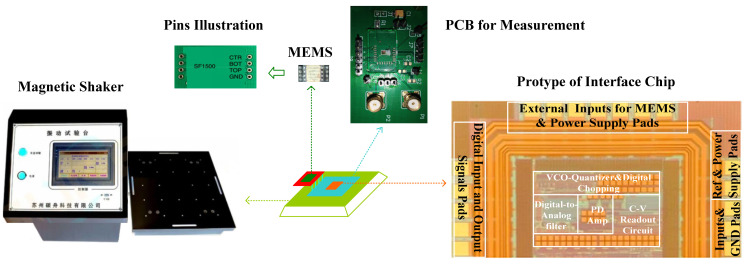
Interface chip protype and the interface measurement set up.

The interface circuit was fabricated in a 0.18 μm CMOS process. MEMS pins illustration and the interface chip protype are shown in Figure 14: The area of the interface chip was 810 × 1210 µm^2^, and the supply voltage for the measurement was 2 V. As shown in Figure 14, the commercial MEMS SF1500S [19] and the interface were integrated on a PCB for the measurement. The PCB was placed on a magnetic shaker. The magnetic shaker was used to generate the force input signal.

**Figure 15:**
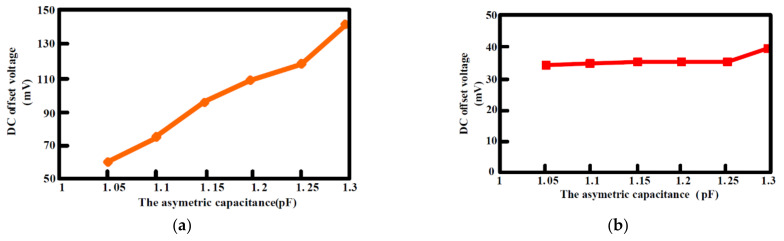
DC output voltage versus the asymmetric capacitance (**a**) without the digital chopping feedback loop; (**b**) with the digital chopping feedback loop.

**Figure 16:**
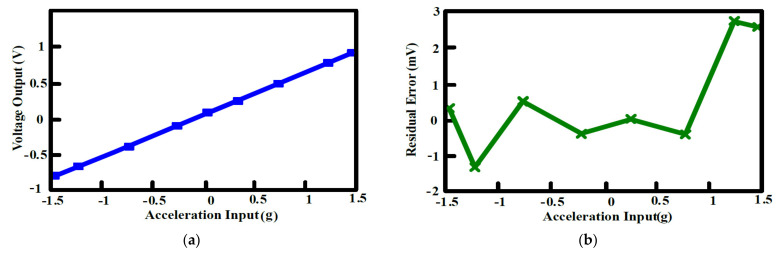
DC acceleration sweep measured (**a**) Voltage output versus acceleration input; (**b**) residual error.

**Figure 17:**
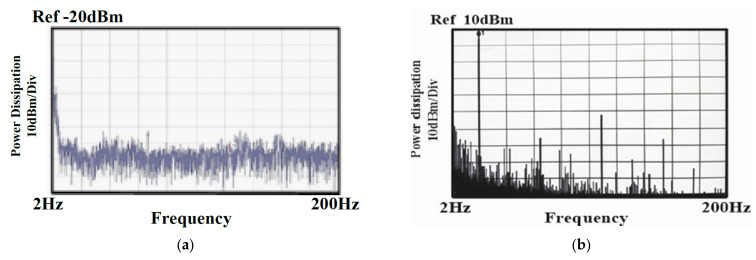
(**a**) Noise Measurement; (**b**) THD Measurement.

**Figure 18:**
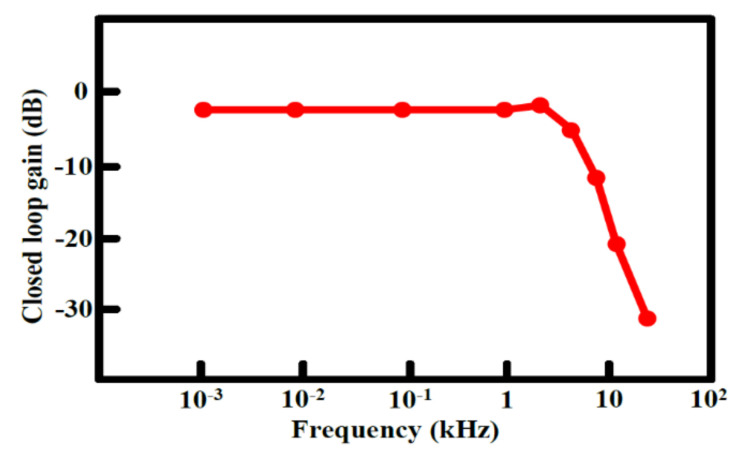
Measured bandwidth of the accelerometer.

The output offset suppression measurement was given as follows: The input referred offset was controlled by designing the two hold capacitors (C_hold_) different (ΔC_hold_ = 1~1.3 pF). The offset was measured without and with the offset cancellation loop operating, as shown in Figure 15a,b: With the loop, the output DC offset voltage was reduced within 40 mV and the output DC offset voltage was almost insusceptible to the variation of the asymmetric parasitic capacitors. The intrinsic output offset may be attributed to the asymmetric capacitors of the accelerometer. The DC characteristic of the input force versus output translating digital signal was measured and the residual error is shown in Figure 16a,b. With the variation from −1.5 g to +1.5 g, the residual error was maintained within 0.38%. The THD was measured at 1 g with a 20 Hz sinusoidal vibration signal. In order to measure the THD of the output signal, the low pass filter using the chip LM324 filtered out the quantization noise in the high frequency range, and AD620 provided the high driving capability and DC ground for the noise and THD measurement. Without the input force, the output noise of the interface is shown in Figure 17a. The equivalent noise of the force achieved 18µV/ sqr Hz. As shown in Figure 17b, the THD of the interface achieved almost −50 dB. The magnitude frequency response of the interface is shown in Figure 18. The band width of the system was about 4 kHz.

Table 1 summarizes and compares the overall system performance with other designs. The table shows that the interface achieved a good dynamic range characteristic, and with the digital chopping nested loop, the DC offset drift could be minimized. Compared to traditional methods, no complicated digital filters or DAC were required for the drift offset minimization. Considering the dynamic range (DR), BW and power performances, the Figure of the Merits (FoM) can be given as follows:

$$FoM=20\mathrm{lg}\left(BW\times DR\right)/\left(Power\times Voltage\right)$$

According to the equation above, the FoM in this work is superior to others, as shown in Table 1.

## 5. Conclusions

This paper describes an output offset minimized capacitance-to-digital interface for an MEMS accelerometer. With a gain-enhanced VCO-based quantizer, the proposed interface directly outputs a digital signal with an improved dynamic range under low supply voltage. With a digital chopping nested loop, the DC offset caused by the bond-wire drift and readout circuit mismatch is minimized. Compared to traditional methods, no digital filters and multibits DAC are required for offset minimization. Therefore, the accelerometer interface achieves lower complexity and cost.

## Figures and Tables

**Table 1:** Performance summary and comparison.

	[3]	[8]	[10]	[14]	[16]	This Work
**Process (μm)**	0.35	0.18	0.35	0.5	0.18	0.18
**Supply (V)**	3.3/15	1.8/3.3	5	7	3	2
Power (mW)	198	0.88	35	23	3	6.5
**Nonlinearity**	0.06%	1.09%	0.16%	0.16%	/	0.38%
**Signal Bandwidth (Hz)**	/	/	1.2 k	0.3 k	1 k	4 k
Full Range	+/−50 g	0–3 g	+/−1.4 g	+/−1.2 g	+/−9 g	+/−1.5 g
**Noise Floor (** **μ** **g/sqr Hz)**	12.7	197	10	0.2	220	18
**Dynamic Range@ (<200 Hz)**	112 dB	64 dB	83 dB	118 dB	75 dB	78 dB
**FoM**	/	/	40	64	59	68
**Drift Offset Suppression**	No	No	No	No	Yes	Yes without Complicated Digital Filters

## Data Availability

The data presented in this study are available on request from the corresponding author. The data are not publicly available due to commercial interests.
